# Mobile Apps for HIV and Sexually Transmitted Infection Prevention in Canada, Mexico, and the United States: Environmental Scan

**DOI:** 10.2196/72009

**Published:** 2025-11-14

**Authors:** Higinio Fernández-Sánchez, Javier Salazar-Alberto, Jhan Carlos Manuel Fernández-Delgado, Annalynn Galvin, Michael J Mugavero, Carlos E Rodriguez-Diaz, Diane Santa Maria

**Affiliations:** 1 Cizik School of Nursing, The University of Texas Health Science Center Houston, 6901 Bertner AvenueHouston, TX, 77030, United States, 7135009921; 2 National Institute of Public Health, Estado de Mexico, Mexico; 3 National University of Cajamarca, Jaén, Peru; 4 The University of Texas Health Science Center Houston, Houston, TX, United States; 5 University of Alabama at Birmingham, Birmingham, AL, United States; 6 Boston University School of Public Health, Boston, MA, United States

**Keywords:** mobile health, mobile app, sexual health, HIV, MARS, Mobile App Rating Scale, STI, sexually transmitted infections

## Abstract

**Background:**

Canada, Mexico, and the United States are primary transit destinations for migrants in the Western Hemisphere. Migrants face barriers to accessing health services, including HIV and AIDS and sexually transmitted infection (STI) prevention. Mobile apps may enhance public health access for these populations.

**Objective:**

This study aims to systematically identify and evaluate mobile apps supporting HIV and STI prevention in Canada, Mexico, and the United States.

**Methods:**

An environmental scan of 357 mobile apps from the Google Play and Apple App stores was conducted on June 18, 2024, following the rigorous 6-step framework proposed by Fernández-Sánchez to ensure a systematic and comprehensive evaluation of apps for HIV and STI prevention. Predefined inclusion and exclusion criteria were applied, resulting in 6 eligible apps. Each app was assessed using the 29-item Mobile App Rating Scale (MARS), scored on a 5-point Likert scale (1=inadequate, 5=excellent), and categorized as high (3), medium (2), or low (1) based on mean scores. Internal consistency was excellent (Cronbach α=0.90), and interrater reliability demonstrated near-perfect agreement (Cohen κ=0.862). Data analyses were performed using SPSS (version 27; IBM Corp).

**Results:**

All 6 apps were available in Canada, Mexico, and the United States, with 33.3% (2/6) from Google Play, 16.7% (1/6) from Apple, and 50% (3/6) from both platforms. MARS evaluation revealed high quality ratings for engagement (83.0%), functionality (88.9%), aesthetics (83.3%), and information quality (100%), as well as high subjective quality (83.3%) and app-specific quality (88.9%). Life4Me+ was the highest-rated app (4.6), while HIV-TEST received the lowest rating (3.4). Most apps (5/6, 83.3%) were only available in English, and 16.7% (1/6) supported multiple languages, which may limit accessibility for non–English-speaking migrant populations. In addition, 83.3% (5/6) were updated in 2024, 33.3% (2/6) were linked to nongovernmental organization, 16.7% (1/6) to a university, and 50% (3/6) had no clear affiliation. Regarding their focus, 50% (3/6) addressed STI prevention, diagnosis, and treatment, 16.7% (1/6) combined HIV and STI prevention, and 33.3% (2/6) provided pre-exposure prophylaxis–related resources.

**Conclusions:**

These 6 apps stand out for their high functionality, engagement, and accessibility, establishing themselves as effective tools for HIV and STI prevention education among migrant populations. This study highlights the critical role of digital resources in addressing public health challenges faced by vulnerable and minority groups. Integrating these apps into health promotion strategies is essential to improve health literacy and encourage preventive behaviors. Moreover, ensuring the quality, credibility, linguistic diversity, and continuous updating of these digital interventions is crucial to achieving a real and sustained impact on public health. Policies should promote clear standards that guarantee accessibility, transparency, and accuracy, thereby facilitating access to health care services in complex migratory contexts.

## Introduction

### Background

Migration to North America is one of the most active migration routes globally [1]. Latin America plays a central role in this dynamic, as Mexico is the largest source of migrants to the United States [2,3]. Other Latin American nations also contribute to migration flows toward Canada, the United States, and Mexico, as these regions serve as key transit and destination points for diverse migrant populations. While the United States remains the primary destination owing to perceived economic and social opportunities [4], increasingly restrictive immigration policies have led to heightened detentions and deportations. Canada has emerged as an alternative destination, but accessibility challenges and strict migration controls complicate pathways, compelling migrants to adapt their routes based on shifting opportunities and policy restrictions [5].

This evolving migration landscape introduces critical public health challenges, as migrants face substantial barriers to accessing health care and are at increased risk for infectious diseases, including HIV and sexually transmitted infections (STIs) [6]. Without adequate prevention and treatment, HIV progresses to AIDS, significantly impacting individual health, quality of life, and the capacity of health care systems in host countries [7]. For migrants, vulnerabilities such as undocumented status, limited health care access, and cultural stigmas exacerbate their susceptibility to HIV and STIs and their negative outcomes [8-10].

The socioeconomic conditions of many migrants, characterized by poverty, instability, and precarious living arrangements, further heighten their exposure to risk factors, including sexual violence, human trafficking, and substance and alcohol use [11-13]. These vulnerabilities not only undermine individual health but also contribute to the transmission of HIV, with significant public health implications for both migrant communities and their host countries [14,15]. The compounded risks underscore the urgent need for effective HIV and STI prevention strategies that can reach these transient populations.

Innovations such as pre-exposure prophylaxis (PrEP), nonoccupational postexposure prophylaxis, postexposure prophylaxis, doxycycline postexposure prophylaxis, and self-testing kits have revolutionized HIV and STI prevention. However, migrants often encounter barriers that limit their access to these tools, including linguistic and cultural challenges [16]. Mobile health (mHealth) apps have emerged as promising platforms to address these barriers, providing accessible, discreet, and culturally relevant health information [17]. Evidence suggests that mHealth apps dedicated to prevention can enhance access to information, especially for minoritized populations such as migrants and others, racially and ethnically diverse groups, as found in recent randomized controlled trials of HIV prevention apps [18-21]. These apps facilitate equitable health care access by offering content in native languages and by integrating functionalities such as testing reminders and treatment adherence tracking [22].

Despite the potential of these apps, ensuring the quality and reliability of the information they provide remains challenging, particularly among migrant populations [23]. Current app stores, such as Google Play and Apple App Store, host numerous health-related apps. Yet only some are designed to meet the specific needs of racially and ethnically diverse populations. For instance, a review of 55 HIV and STI prevention apps found that only 9 catered to racialized or ethnically diverse groups [24]. This limited scope highlights the pressing need to evaluate app quality, participant acceptability, and language appropriateness to ensure these tools effectively support prevention efforts for marginalized groups.

### Objectives

To address this gap, this study used an environmental scan (E-scan) methodology to systematically identify and evaluate mobile apps supporting HIV and STI prevention in Canada, Mexico, and the United States. The E-scan methodology enables a comprehensive analysis of app features, accessibility, and information quality, offering a structured and innovative approach to app evaluation [25]. Given the lack of regulation in health app content [26], this study fills a critical void by evaluating the quality of digital resources tailored to migrant populations. Such research can help establish a foundation for leveraging mHealth tools to enhance HIV and STI prevention across North America, bridging the gap between technological innovation and public health needs. The objective of this study was to systematically identify and evaluate mobile apps that support HIV and STI prevention in Canada, Mexico, and the United States.

## Methods

### Study Design

We conducted an environmental scan (E-scan) following the 6 steps proposed by Fernández-Sánchez et al [25], to systematically identify, screen, and analyze mobile apps available for HIV and STI prevention as of June 18, 2024. This method was combined with PRISMA (Preferred Reporting Items for Systematic Reviews and Meta-Analyses) 2020 guidelines to document the identification, screening, eligibility, and inclusion process through a standardized flow diagram.

### Step I: Research Questions

Our research focused on the following overarching question: What is the quality (ie, engagement, functionality, aesthetics, and information quality) of mobile apps designed for HIV and STI prevention in Canada, Mexico, and the United States? These criteria were selected based on the Mobile App Rating Scale (MARS) [27]. In addition, the study addressed several sub-questions to provide a comprehensive evaluation of the mobile apps: (1) What is the quality of these apps according to user criteria (ie, app subjective quality)? (2) Do these apps allow for user input, feedback, and other prompts (ie, app interactivity)? and (3) How accurate is the HIV and STI prevention information on the app (ie, app-specific quality*)*? Other considerations included the number of languages each app supports, the countries where they are available, the target age range, and the year of their most recent update. Combined, these aspects provided a broader understanding of the apps’ usability, accessibility, and relevance to diverse user needs.

### Step II: Selection Criteria

To answer these research questions, we included mobile apps that were (1) related to HIV and STI prevention and (2) available in Canada, the United States, and Mexico. To broaden the scope of the search, we did not exclude apps-based release date, cost, number of downloads, or the digital medium. We excluded apps developed for health care professionals and not for a public audience.

### Step III: Search Strategy and Data Sources

We thoroughly searched the Google Play Store and Apple App Store by accessing the platforms in all 3 countries. The search process was developed following the PRISMA 2020 guidelines to ensure methodological rigor, transparency, and reproducibility to ensure organized selection and a clear summary of findings [28]. Overall, 3 team members created accounts in the Google Play Store and Apple App Store in Canada, Mexico, and the United States. Apps were identified from May 23 to June 18, 2024, using mobile devices, computers, and iPads with Android and iOS operating systems. A manual search was also conducted on Google to identify potential complementary apps. Keywords in Spanish, English, and French were based on recommendations from similar articles and the authors’ experience. These keywords included: “HIV,” “AIDS,” “PrEP,” “human immunodeficiency virus,” “acquired immunodeficiency syndrome,” “HIV prevention,” “AIDS prevention,” “HIV education,” “HIV/AIDS,” “HIV treatment,” “HIV app,” “HIV health,” “STIs,” “sexually transmitted infection,” “STI prevention,” “STI app,” “sexual health,” “STI education,” and “STI treatment.” Boolean operators (AND, OR) were used to combine keywords and maximize search coverage effectively. The first 100 results of each keyword combination were reviewed, considering that limited time and few human resources were available.

### Step IV: Data Extraction

Data were extracted via Microsoft Excel. The research team designed an Excel table aligned with the purpose of this E-scan to collect relevant information about the apps, including the app name, developer contact information, digital store type, rating, number of downloads, dates of release and last update, version, cost, focus and brief description, theoretical background or strategies, affiliation, target age group, and technical aspects. Data extraction was independently performed by 2 reviewers from the team (JS-A and JCMF-D). Discrepancies were resolved by consensus, and a third reviewer, the principal investigator, validated the data.

### Step V: Quality Assessment

The selected apps underwent a quality assessment using the MARS, developed by Stoyanov et al [29]. The MARS is a multidimensional, reliable, and customizable app quality assessment tool, suitable for health researchers. A team of 3 researchers with quantitative experience consulted articles on the use of the MARS, which are fundamental for avoiding deviations in the quantitative research process [30]. The test-completion procedures were carried out with the principal investigator to gain an initial understanding of the MARS evaluation instrument. During the anchoring process, the use of basic concepts for completing the instrument was standardized to ensure clarity and effectiveness in data collection [31]. Subsequently, the apps were divided for peer evaluation, and once 2 evaluations were obtained, averages were calculated for use. The scale demonstrated high internal consistency, with a Cronbach α coefficient of 0.90. In addition, the subscales (app quality rating, app subjective quality, and app-specific quality) showed strong internal consistency, with a mean coefficient of 0.85 [29].

The MARS uses 3 categories to evaluate several aspects of mobile app quality. The first category, “app quality rating,” is assessed across 4 dimensions [27]: engagement (5 items: fun, interest, individual adaptability, interactivity, and target group), functionality (4 items: performance, usability, navigation, and gestural design), aesthetics (3 items: layout, graphics, and visual appeal), and information quality (7 items: accuracy of app description, goals, quality of information, quantity of information, quality of visual information, credibility, and evidence base). The second category, app subjective quality, comprises 4 questions (ie, “What is your overall star rating of the app?”). The third category, app-specific quality, comprises 6 questions (ie, “Is app content correct, well-written, and relevant to the goal/topic of the app?”)*.* All items were rated on a 5-point scale*,* where 1 indicates “inadequate,” 2 indicates “poor,” 3 indicates “acceptable,” 4 indicates “good,” and 5 indicates “excellent.” Thus, the scale contained 29 items that enabled a comprehensive and accurate assessment of mobile app quality.

For scoring, points were summed for each subscale, and tertile categories (low, medium, and high) were constructed in Microsoft Excel, considering the minimum and maximum scores possible and their ranges. This allowed for precise categorization of quality levels. For the first category, app quality rating, in the engagement subscale, 3 levels were defined: low (5 to 11 points), medium (12 to 18 points), and high (19 to 25 points). For functionality, the levels were: low (4 to 9 points), medium (10 to 14 points), and high (15 to 20 points). In terms of aesthetics, levels were classified as low (3 to 7 points), medium (8 to 11 points), and high (12 to 15 points). Finally, for information, the levels were: low (7 to 16 points), medium (17 to 25 points), and high (26 to 35 points). The second category, app subjective quality, had the following levels: low (4 to 9 points), medium (10 to 14 points), and high (15 to 20 points). Finally, in the third category, app-specific quality, levels were defined as low (6 to 14 points), medium (15 to 22 points), and high (23 to 30 points).

### Step VI: Analysis and Synthesis

Based on the mean obtained by the app evaluators, values per level were assigned for each app as evaluation categories. These values were coded on a scale of 1 to 3, where high corresponds to 3 points, medium to 2 points, and low to 1 point. Percentages were established based on the criteria and evaluated apps. Two reviewers (JS-A and JCMF-D) independently conducted the assessment, resolving discrepancies by consensus. Results were processed using SPSS v27, and Cohen κ was used to measure inter-rater agreement [32]. The calculated Cohen κ coefficient was 0.862, indicating near-perfect agreement between the evaluators. Descriptive statistics, frequencies, and percentages were used for data analysis. The MARS results are presented through stacked bar graphs and box plots. Directed content analysis was applied to narrative information, identifying patterns related to HIV and STI prevention methods. This methodology ensures a comprehensive and accurate evaluation of mobile apps for HIV and STI prevention, providing a solid foundation for future research and public health practice.

## Results

### General Characteristics of the Final Apps

**Figure 1.**
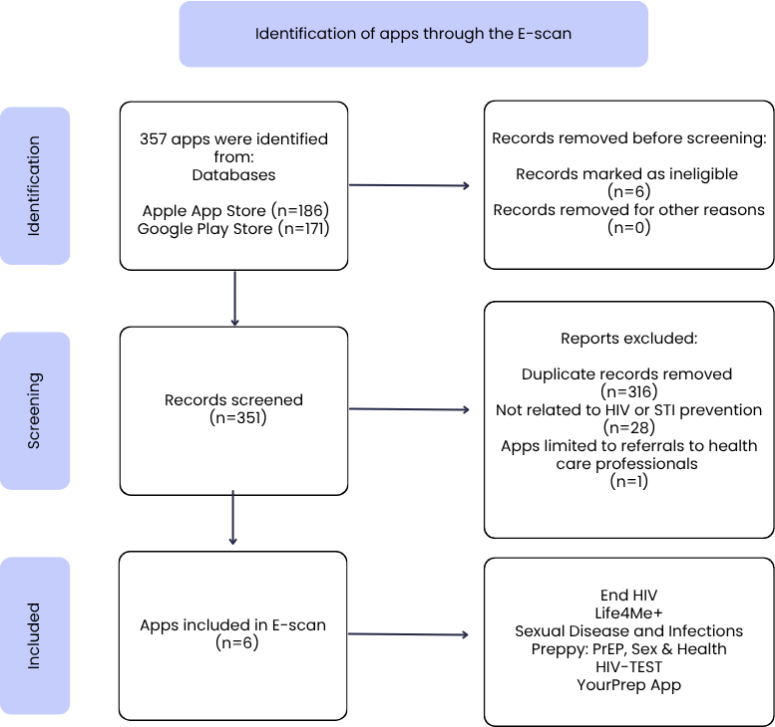
PRISMA (Preferred Reporting Items for Systematic Reviews and Meta-Analyses) flow diagram for included apps. E-scan: environmental scan; STI: sexually transmitted infection.

Figure 1 shows the PRISMA flowchart. The search identified a total of 357 apps, of which 186 were from the Apple App Store and 171 from the Google Play Store. The distribution by country was as follows: an average of 78 were available in Canada, 148 in the United States, and 131 in Mexico (overall mean 119, SD 36.52). The detailed organization of the apps facilitated selection by country, with independent review by JSA, JCMFD, and the principal investigator. As a result, 351 screened apps were eliminated, leaving 6 apps included in this review: “End HIV,” “Life4Me+,” “Sexual Disease and Infections,” “Preppy: PrEP, Sex & Health,” “HIV-TEST,” and “Your Prep App.”

All 6 identified mobile apps were available on either the Apple App Store (1/6, 16.7%), Google Play Store (2/6, 33.3%), or both platforms (3/6, 50%). Overall, 5 of the 6 apps (5/6, 83.3%) were available exclusively in English, while 1 app (1/6, 16.7%) supported 25 languages, including Spanish (Life4Me+). Most of the apps (5/6, 83.3%) had been updated in 2024, with the remaining 1 app (1/6, 16.7%) last updated in 2023. Two apps (2/6, 33.3%) were linked to nongovernmental organizations, 1 app (1/6, 16.7%) was linked to a university, and 3 apps (3/6, 50%) had no clearly identified affiliations. Most apps (5/6, 83.3%) were designed for individuals 17 years and older, while 1 app (1/6, 16.7%) was reported to be suitable for users 12 years and older (End HIV). The apps shared common objectives, including promoting PrEP, facilitating HIV testing, providing HIV risk assessment tools, and supporting HIV/STI treatment. Their specific focuses on HIV and STI prevention and care were categorized as follows: ( 1) prevention, diagnosis, and treatment of STIs (3/6, 50%); (2) combined HIV and STI prevention (1/6, 16.7%); and (3) PrEP-related resources (2/6, 33.3%) (Table 1).

**Table 1.** Characteristics of HIV and sexually transmitted infection (STI) prevention mobile apps in Canada, Mexico, and the United States.

App number and name	Platform (Android and iOS), languages, privacy, customization, and connectivity.	Developer	Goals	Approach	Year of update	Tested app version	Mean User Star Rating^a^	MARS^b^ app quality rating score, mean (SD)^a^	MARS app subjective quality score, mean (SD)^a^	MARS app-specific quality, mean (SD)^a^
End HIV	Both, English, does not share data, and it is personalized, free.	University of Mississippi Medical Center	Inform about HIV prevention, testing, PrEP^c^, and health care	HIV prevention, diagnosis, and treatment	2024	2.1.0	—^d^	4.55 (0.11)	3.9 (0.40)	4.5 (0.17)
Life4Me+	Both, English, Spanish, and 16 languages, does not share data, and is personalized, free.	Life4me.plus fight to AIDS, Hepatitis C, and Tuberculosis	Prevents HIV, or people already living with it, as well as other STIs	HIV prevention, STI prevention	2024	2.0.40	—	4.68 (0.13)	4.5 (0.10)	4.4 (0.22)
Sexual Disease and Infections	Android, English, does not share data, and is personalized, free.	Encoded Knowledge Ltd. Silversky Technology	Provide information about STDs^e^, including symptoms, treatments, and prevention	STI prevention, diagnosis, and treatment	2024	1.25	5	4.6 (0.22)	3.5 (0.25)	3.3 (0.34)
Preppy: PrEP, Sex & Health	Both, English, does not share data, and is personalized free.	Prepsafe Global	Helps users manage PrEP, their sexual health, and well-being	Pre-exposure prevention	2024	1.2.3	4.6	4.57 (0.13)	4.1 (0.17)	4.5 (0.16)
HIV-TEST	Android, English, does not share data, and is personalized free.	Merezha	Provide a risk measurement tool that estimates the probability of HIV infection	HIV prevention	2024	2.3.1	—	4.18 (0.14)	3.1 (0.38)	4.3 (0.21)
YourPrEP App	Android, English, does not share data, and is personalized, free.	GGD Amsterdam	Inform about the use of PrEP	Pre-exposure prevention	2023	1.0.3.51	—	4.47 (0.10)	3.1 (0.32)	4.3 (0.17)

a Overall mean values are 4.8 (SD 0.28) for Mean User Star Rating, 4.5 (SD 0.17) for Mobile App Rating Scale (MARS) app quality score, 3.7 (SD 0.56) for MARS app subjective quality score, and 4.4 (SD 0.08) for app-specific quality.

b MARS: Mobile App Rating Scale.

c PrEP: pre-exposure prophylaxis.

d Not available.

e STD: sexually transmitted disease.

### Overall App Quality Assessment With MARS

**Figure 2.**
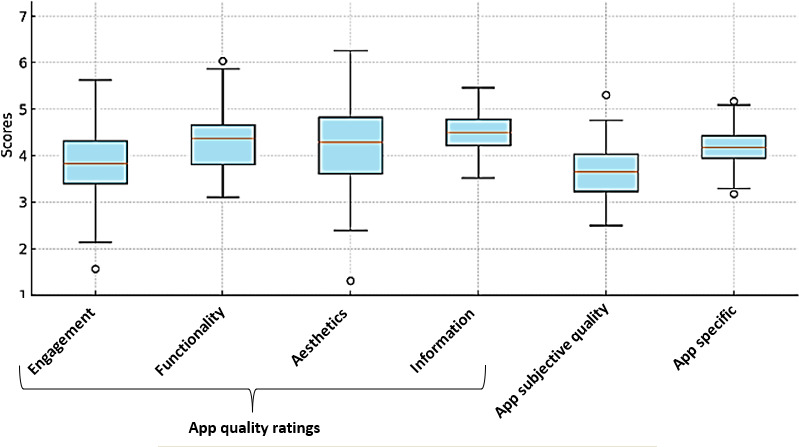
Mobile App Rating Scale (MARS) scores by dimensions.

The overall app quality assessment using the MARS framework revealed positive results across all 4 dimensions. Engagement had a mean score of 3.95 of 5 (SD 0.91), with apps achieving high ratings of 83.0%. Functionality demonstrated a mean of 4.32 (SD 0.63), with apps scoring highly (88.9%). Aesthetics achieved a mean score of 4.20, with apps receiving high ratings of 83.3%. The information dimension had the highest mean score of 4.48 (SD 0.45), with the apps scoring highly (100%). Among the 6 highest-rated apps, Preppy: PrEP, Sex & Health scored highest in engagement (4.8/5), End HIV excelled in functionality (4.9/5), Sexual Disease and Infections led in aesthetics (4.9/5), and the YourPrEP app received a perfect score in the information dimension (5/5) (Figure 2).

**Figure 3.**
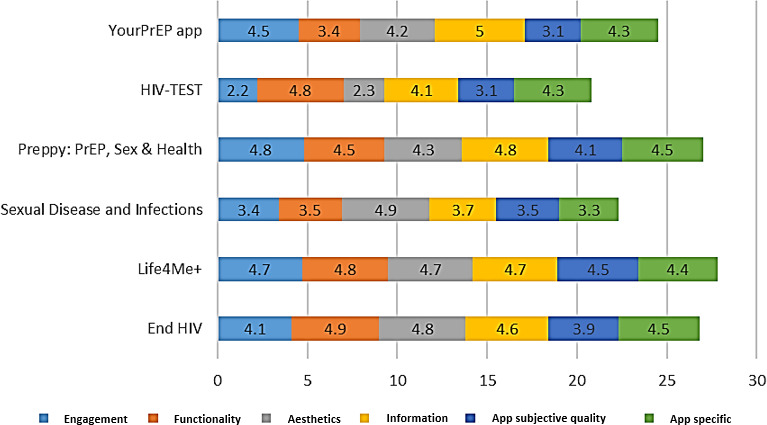
Cumulative score of each app’s Mobile App Rating Scale (MARS) subdomains.

The subjective quality of the apps also yielded favorable results, with a mean score of 3.70 (SD 0.52) and the apps receiving high ratings of 83.3%. The highest subjective quality score was recorded for Life4Me+ (4.5/5), while the lowest ratings were observed for HIV-TEST and the YourPrEP app, both scoring 3.1/5. Similarly, the app-specific quality assessment revealed a mean score of 4.22 (SD 0.42), with apps achieving high ratings of 88.9%. The highest scores in this category were shared by Preppy: PrEP, Sex & Health, and End HIV, both averaging 4.5/5, while Sexual Disease and Infections received the lowest score (3.3/5). Overall, the results highlight the strong performance of these apps across multiple quality dimensions, underscoring their potential use in addressing HIV prevention and care (Figure 3).

### Barriers and Facilitators

The following are the main barriers and facilitators identified and also the strengths and weaknesses found for each app were addressed (seeMultimedia Appendix 1 and Garcia et al [33]). The relevant aspects of barriers and facilitators were grouped into three sections that are (1) technological, (2) social and psychological, and (3) governance; further details about these barriers are listed below.

#### Technological

##### Technical

Facilitating features included aspects such as the type of operating system and the speed of downloads. The diversity of mobile devices, browsers, and assistive technologies was noted, following platform accessibility guidelines with which the apps are compatible. The apps generally used good visual or auditory design.

##### Content-Related

Facilitating features included the year of creation and update, availability of languages, free access in most cases, and continuous online availability. They used simple language, clear information, and appropriate hierarchy in information distribution, since without these aspects, legibility, comprehension, and usability could be compromised. In this study, all the apps addressed topics related to HIV and STIs to a greater or lesser degree. On the other hand, barriers included some apps requiring user registration for access, though this was less common. A lack of or inconsistency in app metadata (downloads and ratings) was also observed, which may lead to data loss and variation in the classification of information during analysis. Among the studied apps that mentioned HIV and STI detection and treatment, all emphasized that their use does not substitute for physical testing, treatment, or medical consultations, recognizing that use is the responsibility of the user [34]. In addition, there was very little mention of migrant populations, as the apps primarily targeted men who have sex with men [35].

### Social and Psychological

#### Age

This was generally a facilitator, as the apps were understandable for almost all audiences. Most were identified as suitable for users older than 10 years. *Economic:* Mobile phones can serve as facilitators since they are relatively accessible and portable when small. However, they can become barriers if users cannot afford a smartphone, or if the device is larger (such as an iPad, tablet, or laptop), making transport more difficult.

#### Cultural Sensitivity (Stigma)

Facilitating aspects included awareness and respect for diversity, culture, values, beliefs, and personal preferences. Most apps did not request personal information beyond an email account, alias, and confirmation of age. Generally, if personal information was requested, a privacy notice was provided. In this study, people living with HIV were not involved in the app evaluations.

#### Cognitive Characteristics (Ease of Use, Usefulness, Satisfaction, Digital Literacy, Online Participation, and Trust in the Internet)

Facilitating aspects included ease of use. Usefulness and satisfaction were supported by positive user comments, which could serve as suggestions for improvement. Digital literacy was observed through the rapid learning curve when using the apps. Online participation was reflected in the number of stars and downloads, indicating that apps had been marked as highlighted or favorites. However, a main barrier was trust in internet access: without internet connectivity, users cannot download or use the app [36].

### Governance (Legal compliance)

As a facilitating feature, most apps had country-specific but legal permissions. However, this aspect varied, and consultation with legal experts was deemed essential to avoid lawsuits, fines, or damages.

## Discussion

### Principal Findings

Digital interventions offer an inclusive, adaptable, and confidential approach to prevention, contrasting with traditional in-person interventions that are often inaccessible, fear-based, and ineffective for populations made vulnerable [37]. This study focused on mobile apps related to HIV and STI prevention across Canada, Mexico, and the United States. Using the E-Scan methodology, recognized for its reliability in selecting mobile apps [25], the study used the MARS tool to evaluate 6 apps. These apps were assessed based on quality ratings (engagement, functionality, information, and aesthetics), app subjective quality, and app-specific criteria.

All the apps included in this study shared common goals related to HIV and STI prevention. This availability of the service, but at the same time, the limited access for the migrant population underscores a gap in addressing public health issues such as HIV and STI prevention, particularly among vulnerable populations [38,39]. High information and functionality scores reflect intuitive and practical interactions that encourage adoption [29]. However, differences in app quality, such as low interactivity, can impede user engagement and learning [40]. This study revealed high scores for information, with slightly lower engagement scores, highlighting areas for improvement to enhance user satisfaction. Appealing aesthetics further help sustain user attention [41], who noted that app quality significantly influences user trust and decision-making. Despite the proliferation of health apps, concerns remain about the reliability of information for nonprofessional users [42]. Users often face an overwhelming number of options without clear quality indicators owing to a scarcity of scientifically validated evidence underpinning health app content [43]. Evidence-based apps supporting HIV and STI prevention among minoritized populations, including migrants, show promise [44-46] but must overcome structural barriers and implementation costs to increase their reach. Ensuring updated, relevant, and reliable content is critical to maintaining credibility, while subjective and specific quality criteria ensure personalized and trustworthy user experiences.

Understanding user engagement and app scalability is crucial for addressing health access barriers. While most apps in this study were accessible in English, fewer supported other languages. Language availability is essential for reducing barriers to HIV care and prevention [47,48]. Language-specific studies revealed a lack of interventions tailored for Spanish-speaking Latino individuals, adolescents, and men who have sex with men [49]. Accurate translation of HIV and STI information was inconsistently noted in app descriptions, emphasizing the need for linguistic rigor. These findings highlight the importance of leveraging MARS ratings to guide future app development and improvements in accessibility, reach, acceptability, and effectiveness.

### Implications for Practice, Research, and Policy

The findings from the E-Scan and MARS evaluation have critical implications for practice, research, and policy. Practitioners and public health organizations should integrate mobile apps into comprehensive health promotion strategies, particularly for populations made vulnerable, such as migrants. High engagement, functionality, and information quality make these apps valuable tools for enhancing health literacy and promoting preventive behaviors, though addressing aesthetic and subjective quality concerns is necessary to improve user experience and sustain engagement.

Regarding usefulness, given the increasing use of mHealth apps alongside the growth of social networks, smartphones, and advancing technology, these tools should be leveraged and translated into activities focused on HIV and STI prevention, detection, and medical care among migrant and undocumented populations. For example, among young men, several factors are associated with increased risk of infection, such as risky sexual behaviors through dating apps, a history of sexually transmitted infections, sexualized drug use, and pornography consumption, among others [50-52]. These actions can be implemented across multiple settings, since this population is immersed in diverse environments: educational centers (schools and universities), workplaces, government centers (migrant detention centers, immigration embassies, and legal agencies), community centers (shelters, civil society organizations, and faith-based organizations), cultural spaces, and commercial venues, among the most relevant [53]. Efforts should include the distribution and dissemination of flyers, brochures, posters, and wall newspapers, as well as incorporation into pedagogical activities, nursing talks, and health promotion sessions, with explicit mention of the existence of these apps. Strategies from mass media (eg, television, radio, social networks such as Facebook [Meta], Twitter [Meta], and Instagram [Meta]) can also be leveraged.

Undocumented migrant populations are also served in health care institutions, where strategies can be developed across the 3 levels of health care. At the first level of care, with a community-based focus, activities may include communication, health promotion, prevention, and early detection of HIV and STIs, including dissemination of information about the existence of apps [54]. At the second and third levels of care, which are more focused on treatment and referral of newly diagnosed individuals to other services, these activities can be incorporated into health programs and projects (eg, best practices or evidence-based practices). Information about HIV and STI-related apps can also be included on hospital websites and in prevention campaigns delivered by nurses, physicians, social workers, psychologists, and health educators. Finally, at tertiary-level or specialty hospitals that function as research centers, the topic of HIV and apps can be formally integrated into clinical and educational initiatives.

This article underscores the need for ongoing evaluation of digital health tools using frameworks such as MARS to assess their real-world effectiveness. Future studies should refine evaluation tools and methodologies such as the E-Scan to incorporate diverse user experiences and cultural contexts, ensuring broader accessibility and relevance. Ensuring apps adhere to evidence-based practices and remain updated with reliable information is crucial for advancing digital health interventions. Policymakers should prioritize developing guidelines and standards to ensure health apps meet high-quality criteria, focusing on transparency, accuracy, and accessibility [55,56]. Given the increasing barriers to accessing health care, apps with accurate and reliable information can support migrant populations navigating changing political landscapes in search of health care services.

Changes in the political climate in the United States and globally have increased fear among immigrants, leading to missed appointments and relocations [57]. State and local policies across sectors such as labor, health, and education influence HIV vulnerability among Latino migrants by shaping access to institutions [58]. For example, state-level legislation that requires hospitals to collect immigration status may lead to less health care use, as such measures can deter undocumented or mixed-status families from seeking care due to fears of disclosure, discrimination, or legal repercussions. This reduced engagement with health care services can delay HIV testing, prevention, and treatment, thereby increasing vulnerability within migrant communities [59]. Moreover, immigration policies are impacted by partisan polarization and right-wing populism, aspects in which progress is not achieved, such as full access to health care for migrants, the availability of international medical graduates, which are essential for primary care in underserved areas, among other aspects [60]. Language policies addressing social inclusion for migrants are critical to reducing inequality and ensuring access to public services [61].

### Strengths and Limitations

This study systematically evaluated mHealth apps across 3 countries, key destinations for migrants, leveraging the bilingual expertise of its researchers in Spanish and English. The use of 3 reviewers per app and Cohen κ calculations ensured accuracy and consistency. However, limitations include the rapid evolution of health apps, potentially excluding newer options, and challenges in evaluating instruments in languages not native to the researchers. The study also did not incorporate direct user feedback or usability testing, which could provide critical insights into real-world adoption, engagement, and satisfaction. The generalizability of the MARS ratings to undocumented migrants may be limited, as this group may face distinct access barriers and usage patterns that were not directly assessed. In addition, the use of tertiles to interpret MARS scores may be less reliable with a small sample size, potentially limiting the robustness of these categorizations. Another limitation is the lack of comparison between app content and authoritative online resources, such as Centers for Disease Control and Prevention websites, which could help assess the relative quality and completeness of the information provided. Moreover, while this study was unable to assess the efficacy or perception of behavior change resulting from the apps, doing so would be a good next step in their evaluation. Despite these limitations, the study provides a comprehensive view of the availability, accessibility, and quality of HIV and STI prevention apps, emphasizing the need for linguistic accessibility and optimized user experiences.

### Conclusions

The mobile apps analyzed in this study, focusing on HIV and STI prevention, are available in Canada, the United States, and Mexico through major app stores. These apps primarily offer free access and support multiple languages. The MARS evaluation revealed high performance in engagement, functionality, and information quality, with room for improvement in aesthetics and subjective ratings. These findings underscore the critical role of digital resources in addressing public health challenges faced by minoritized populations, such as migrants. Expanding methodologies like the E-Scan and tools like the MARS could further enhance the field. Addressing quality concerns is essential to ensuring that digital interventions provide credible, satisfying, and effective solutions that drive tangible health impacts. High standards in engagement, functionality, and information are not only technical requirements but fundamental necessities for promoting app credibility and effectiveness in public health promotion.

## Supplementary material

10.2196/72009 Multimedia Appendix 1 Table of strengths and weaknesses of each application.

## Abbreviations

E-scan: environmental scan
MARS: Mobile App Rating Scale
PrEP: pre-exposure prophylaxis
PRISMA: Preferred Reporting Items for Systematic Reviews and Meta-Analyses
STI: sexually transmitted infection

## References

[1] Giourguli-Saucedo SE, Lazcano-Ponce E (2024). Migración y salud: el reflejo de la inequidad social global y la necesidad de investigación en salud pública [Article in Spanish]. Salud Publica Mex.

[2] Bojórquez I, Cerecero-García D, Orraca-Romano PP, Fernández-Niño J, Rojas-Botero M, Infante C (2024). Atención en salud a migrantes en tránsito: estimación del costo para el sistema de salud en México [Article in Spanish]. Salud Publica Mex.

[3] Zamora Salazar C, Casillas R (2024). Detención de migrantes indocumentados en Estados Unidos: ¿quién es quién en aprehensiones? [Article in Spanish]. Migr Inter.

[4] (2022). Migraciones sur-norte desde sudamérica: rutas, vulnerabilidades y contextos del tránsito de migrantes extrarregionales [Report in Spanish]. https://lac.iom.int/sites/g/files/tmzbdl626/files/documents/oim_migraciones-sur-norte-desde-suramerica.pdf.

[5] Reitz JG, Hernández Jasso M (2023). Growth in high-skilled Mexican migration northward: American and Canadian destinations [Article in Spanish]. Migr Inter.

[6] Guerra-Ordoñez JA, Benavides-Torres RA, Zapata-Garibay R, Ruiz-Cerino JM, Ávila-Alpirez H, Salazar-Barajas ME (2022). Percepción de riesgo para VIH y sexo seguro en migrantes de la frontera norte de México [Article in Spanish]. Revista Internacional de Andrología.

[7] Parco Gavilánez MC (2023). Calidad de vida de los pacientes VIH positivo [Article in Spanish]. Ciencia Latina.

[8] Swinkels HM, Gulick PG, Nguyen AD (2024). StatPearls [Internet].

[9] (2023). Aumento de la migración en las américas en 2023: retos para garantizar la salud de las personas migrantes y respuesta de la organización panamericana de la salud [Web page in Spanish]. OPS/OMS.

[10] Kaur H, Saad A, Magwood O (2021). Understanding the health and housing experiences of refugees and other migrant populations experiencing homelessness or vulnerable housing: a systematic review using GRADE-CERQual. CMAJ Open.

[11] Zhang L, Au W, Ewesesan R, Yakubovich AR, Brownridge DA, Urquia ML (2024). Intimate partner violence among international and interprovincial migrants: a population-based analysis of Canadian linked immigration and justice data. Violence Against Women.

[12] Sabri B, Njie-Carr VPS, Messing JT (2019). The weWomen and ourCircle randomized controlled trial protocol: a web-based intervention for immigrant, refugee and indigenous women with intimate partner violence experiences. Contemp Clin Trials.

[13] Santoso D, Asfia S, Mello MB (2022). HIV prevalence ratio of international migrants compared to their native-born counterparts: a systematic review and meta-analysis. EClinicalMedicine.

[14] Sanchez MA, Lemp GF, Magis-Rodríguez C, Bravo-García E, Carter S, Ruiz JD (2004). The epidemiology of HIV among Mexican migrants and recent immigrants in California and Mexico. J Acquir Immune Defic Syndr.

[15] Li K, Thaweesee N, Kimmel A, Dorward E, Dam A (2024). Barriers and facilitators to utilizing HIV prevention and treatment services among migrant youth globally: a scoping review. PLOS Glob Public Health.

[16] Benoit JRA, Louie-Poon S, Kauser S, Meherali S (2022). Promoting adolescent sexual and reproductive health in North America using free mobile apps: environmental scan. JMIR Pediatr Parent.

[17] Muñoz D, Gomez J, Andrade KV, Institución Universitaria Antonio José Camacho (2025). Salud móvil (mHealth) en profundidad: un estudio bibliométrico [Article in Spanish]. Espacios.

[18] Cunha-Oliveira A da, Gómez Cantarino S, Santos D (2025). Use of digital technologies to promote sexual health in young adults: a narrative review. Rev Bras Enferm.

[19] Biello KB, Marrow E, Mimiaga MJ, Sullivan P, Hightow-Weidman L, Mayer KH (2019). A mobile-based app (MyChoices) to increase uptake of hiv testing and pre-exposure prophylaxis by young men who have sex with men: protocol for a pilot randomized controlled trial. JMIR Res Protoc.

[20] Jones J, Dominguez K, Stephenson R (2020). A theoretically based mobile app to increase pre-exposure prophylaxis uptake among men who have sex with men: protocol for a randomized controlled trial. JMIR Res Protoc.

[21] Biello KB, Hill-Rorie J, Valente PK (2021). Development and evaluation of a mobile app designed to increase hiv testing and pre-exposure prophylaxis use among young men who have sex with men in the United States: open pilot trial. J Med Internet Res.

[22] Reis L, Pereira M, Silveira C (2023). Handbook of Research on Solving Societal Challenges Through Sustainability-Oriented Innovation.

[23] Velázquez Hidalgo AS, Fernández Pérez Y, Pacheco Jeréz YS, Zulueta Véliz Y (2023). Métodos y criterios para valorar la calidad en uso de las aplicaciones Móviles para atención al ciudadano cubano [Article in Spanish]. Rev Cubana Cienc Inform.

[24] Bilal A, Mirza HT, Hussain I, Ahmad A (2024). Investigating influence of Google-Play application titles on success. Big Data Research.

[25] Fernández-Sánchez H, Guerrero-Castañeda RF, Jones J, King K (2024). Exploraciones ambientales en ciencias de la salud [Article in Spanish]. Horiz Enferm.

[26] Harris B, Brooker J (2025). Environmental scanning: a look to the future [Article in Spanish]. New Drctns Evaluation.

[27] Bekar Adiguzel M, Cengiz MA (2023). Model selection in multivariate adaptive regressions splines (MARS) using alternative information criteria. Heliyon.

[28] Page MJ, McKenzie JE, Bossuyt PM (2021). The PRISMA 2020 statement: an updated guideline for reporting systematic reviews. J Clin Epidemiol.

[29] Stoyanov SR, Hides L, Kavanagh DJ, Wilson H (2016). Development and validation of the user version of the Mobile Application Rating Scale (uMARS). JMIR Mhealth Uhealth.

[30] Delgado-Morales C, Duarte-Hueros A Una Revisión sistemática de instrumentos que evalúan la calidad de aplicaciones móviles de salud [Article in Spanish]. Pixel-Bit.

[31] Márquez JL (2023). ¿Somos predeciblemente racionales o predeciblemente irracionales? Un estudio sobre el “efecto anclaje” [Article in Spanish]. ddee.

[32] Duarte-Anselmi G, Leiva-Pinto E, Vanegas-López J, Thomas-Lange J (2022). Experiences and perceptions on sexuality, risk and STI/HIV prevention campaigns by university students. designing a digital intervention. Cien Saude Colet.

[33] Garcia Saiso S, Marti MC, Malek Pascha V (2021). Implementation of telemedicine in the Americas: barriers and facilitators [Article in Spanish]. Rev Panam Salud Publica.

[34] Schaab BL, Remor E (2023). Development, feasibility testing and perceived benefits of a new app to help with adherence to antiretroviral therapy in people living with HIV in Brazil. Pilot Feasibility Stud.

[35] Pravosud V, Ballard AM, Holloway IW, Young AM (2024). Latent class analysis of online platforms for partner-seeking and sexual behaviors among men who have sex with men from Central Kentucky. AIDS Behav.

[36] Mola DJ, Josefina F, Sonia D, Cecilia R Barreras y facilitadores en el uso de las herramientas digitales de gobierno: resultados preliminares [Article in Spanish]. https://ri.conicet.gov.ar/handle/11336/244461.

[37] Blamey R, Sciaraffia A, Piñera C (2024). Situación epidemiológica de VIH a nivel global y nacional: puesta al día [Article in Spanish]. Rev chil infectol.

[38] Rojas D (2023). Acceso y uso de datos móviles en poblaciones migrantes [Web page in Spanish]. Centro LATAM Digital.

[39] Sullivan PS, Hightow-Weidman L (2021). Mobile apps for HIV prevention: how do they contribute to our epidemic response for adolescents and young adults?. Mhealth.

[40] Acosta Espinoza JL, León Yacelga LR, Sanafria Michilena WG (2022). Las aplicaciones móviles y su impacto en la sociedad [Article in Spanish]. Rev Univ Soc.

[41] Preciado-Ortiz CL (2021). Quality and use of mobile applications for transportation service: influence on satisfaction. MYN.

[42] Tala Á, Vásquez E, Rojas E, Marín R (2022). Apps y Medicina: una visión global y la situación chilena [Article in Spanish]. Rev méd Chile.

[43] Collado-Borrell R, Escudero-Vilaplana V, Narrillos-Moraza Á, Villanueva-Bueno C, Herranz-Alonso A, Sanjurjo-Sáez M (2022). Patient-reported outcomes and mobile applications. A review of their impact on patients’ health outcomes. Farm Hosp.

[44] Curioso WH, Blas MM, Kurth AE, Klausner JD (2007). Tecnologías de información y comunicación para la prevención y control de la infección por el VIH y otras ITS [Article in Spanish]. Rev Peru Med Exp Salud Publica.

[45] Santa Maria D, Padhye N, Businelle M (2021). Efficacy of a just-in-time adaptive intervention to promote HIV risk reduction behaviors among young adults experiencing homelessness: pilot randomized controlled trial. J Med Internet Res.

[46] Nahum-Shani I, Naar S (2023). Digital adaptive behavioral interventions to improve HIV prevention and care: innovations in intervention approach and experimental design. Curr HIV/AIDS Rep.

[47] Mounkoro I (2024). Revolucionando el aprendizaje de idiomas: el impacto transformador de las aplicaciones móviles [Article in Spanish]. Apertura.

[48] Marzan-Rodriguez M, Rodriguez-Diaz CE, Mustanski B (2021). Recommendations for the development of HIV prevention interventions among Latino young sexual minority groups. Sex Res Social Policy.

[49] Border health and migration. Centers for Disease Control and Prevention.

[50] Naranjo-Márquez M, Bocchino A, Gilart E, Cotobal-Calvo EM, Procentese F, Palazón-Fernández JL (2025). Risk determinants of sexual behaviors: dating apps, history of sexually transmitted infections, substance use, and pornography consumption in health science students. Nurs Rep.

[51] Marqués-Sánchez P, Bermejo-Martínez D, Quiroga Sánchez E, Calvo-Ayuso N, Liébana-Presa C, Benítez-Andrades JA (2023). Men who have sex with men: an approach to social network analysis. Public Health Nurs.

[52] Lisboa C, Stuardo V, Folch C (2023). Sexualized drug use among gay men and other men who have sex with men in Latin America: a description of the phenomenon based on the results of LAMIS-2018. PLoS ONE.

[53] Rafful C, Orozco R, Peralta D (2024). Feasibility, acceptability, and perceived usefulness of a community-evidence-based harm reduction intervention for sexualized stimulant use among Mexican gay, bisexual, and other men who have sex with men. Harm Reduct J.

[54] González Martínez MÁ, Castaño Suero MJ, Guerrero Muñoz M, Francisco Rossetti A, Sequeira Aymar E, Roca Saumell C (2024). Initial assessment of immigrant patients in primary care. Aten Primaria.

[55] Savas ST, Knipper M, Duclos D, Sharma E, Ugarte-Gurrutxaga MI, Blanchet K (2024). Migrant-sensitive healthcare in Europe: advancing health equity through accessibility, acceptability, quality, and trust. Lancet Reg Health Eur.

[56] Tummala-Narra P (2022). El miedo de los inmigrantes [Article in Spanish]. Aperturas Psicoanaliticas.

[57] Pérez CS (2025). Sistemas de salud y políticas de atención al migrante [Article in Spanish]. FMC - Formación Médica Continuada en Atención Primaria.

[58] (2024). Executive order GA-46 reporting for collection of information on hospital costs related to immigration status. Texas Health and Human Services.

[59] Conozca sus derechos a obtener atención médica y seguro médico [Web page in Spanish]. National Immigration Law Center.

[60] González Delgado D (2024). La política migratoria de Estados Unidos en el siglo XXI: evolución y desafíos [Article in Spanish]. Novedades en Población.

[61] Gazzola M, Wickström BA, Fettes M (2023). Towards an index of linguistic justice. Politics, Philosophy & Economics.

