# Supplementary material for: Mobile Apps for HIV and Sexually Transmitted Infection Prevention in Canada, Mexico, and the United States: Environmental Scan
Source: JMIR Mhealth Uhealth. 2025 Nov 14;13:e72009. doi: 10.2196/72009 (PMC12617829; doi:10.2196/72009)
Supplement: Multimedia Appendix 1 [file mhealth-v13-e72009-s001.docx]

**Appendix**

**Table. S1 Strengths and Weaknesses of Each App**

| **Strengths and Weaknesses of Each App** | | |
| --- | --- | --- |
| **Apps** | **Strengths** | **Weaknesses** |
| **1. End HIV:** | A comprehensive tool providing information on HIV prevention, testing, PrEP, and medical care for people living with HIV. | Notes that the information provided should not be used to diagnose or treat diseases; individuals seeking advice should consult a certified physician. Not primarily focused on STIs. |
| **2. Life4Me+:** | A very comprehensive tool that informs about HIV prevention and provides newly diagnosed individuals with information on how to manage HIV/STIs. Functions as a reminder for testing and medication intake. Facilitates communication by obtaining prescriptions and appointments, with users able to enter their test results into the app. GPS navigation helps locate nearby hospitals, pharmacies, laboratories, or global HIV organizations. Includes regional hotlines helping. | None noted beyond its primary HIV focus. |
| **3. Sexual Disease and Infections:** | Provides educational information about STIs, including symptoms, treatments, prevention, and complications. Available in more than 25 languages. | Not primarily focused on HIV. |
| **4. Preppy: PrEP, Sex & Health:** | Helps users manage PrEP, sexual health, and well-being. Provides information on daily or on-demand dosing, PrEP intake over the last 90 days, calendar, intake history, and smart reminders. | States that it does not replace contact with a healthcare professional; intended for personal use only; PrEP alone does not protect against HIV infection; use is at one’s own risk. Not primarily focused on STIs. |
| **5. HIV-TEST:** | Preventive tool that estimates HIV infection probability through a risk measurement system. Designed to improve HIV testing effectiveness by answering three questions about sex, blood, and symptoms. Based on the answers, the algorithm provides a probability estimate. Includes a locator for the nearest site to obtain a confidential physical test. | Does not provide diagnosis, and accuracy depends on the honesty of user responses. A certified test is recommended for reliable results. Not primarily focused on STIs. |
| **6. YourPrEP App:** | Primarily informs about PrEP use. Functions as a reminder. Targets men who have sex with men, transgender people, and others who have unprotected anal sex with partners of unknown HIV status. | Not primarily focused on HIV/STIs. |
